# Cefazolin-associated coagulopathy in a critically Ill patient with MSSA bacteremia: a case report

**DOI:** 10.1017/ash.2026.10893

**Published:** 2026-10-07

**Authors:** Dakota Sicignano, Julie Gray

**Affiliations:** Yale New Haven Hospital, USA

## Abstract

**Objective:**

We report a case of cefazolin-induced coagulopathy in a patient with a methicillin-sensitive *Staphylococcus aureus* (MSSA) bacteremia.

**Setting:**

Medical intensive care unit at a 1,500-bed, tertiary medical center.

**Design:**

A 74-year-old male presented to the medical intensive care unit with septic shock secondary to a left skin and soft tissue infection. Blood cultures resulted positive for and the patient was initiated on cefazolin therapy. Shortly after initiation of therapy, patient lab work showed an increased INR to a max of >9.67. Hematology was consulted, and further work-up revealed a clotting deficiency consistent with a vitamin K-mediated coagulopathy. Intravenous vitamin K was administered, and the patient had rapid correction of their INR. Therapy was switched to oxacillin for the completion of treatment with no further cases of coagulopathy.

**Conclusion:**

Cefazolin-associated coagulopathy, though rare, should be considered in patients with unexplained INR elevation during therapy. Early recognition is critical, as discontinuation of the offending agent and administration of vitamin K can rapidly reverse coagulopathy. Clinicians should maintain heightened awareness in critically ill patients, particularly when additional risk factors for coagulopathy are present.

## Introduction

Cefazolin, a first-generation cephalosporin, is frequently used for the treatment of skin and soft tissue infections and methicillin-sensitive *Staphylococcus aureus* (MSSA) bacteremia. Cefazolin exerts its effect by inhibiting bacterial cell wall synthesis via penicillin-binding proteins. Although cephalosporin-associated coagulopathy has historically been linked to agents containing an N-methylthiotetrazole (NMTT) side chain, cefazolin lacks this structure. Instead, it contains a methyl-thiadiazole (MTD) side chain. Inhibition of vitamin K epoxide reductase by MTD-containing agents has been demonstrated in animal models and is hypothesized to occur in humans, resulting in reduced synthesis of vitamin K-dependent clotting factors (II, VII, IX, and X).^1,2^ Cefazolin is renally eliminated with a serum half-life that is prolonged in patients with renal dysfunction. Reduced renal clearance has been identified as a risk factor for elevated drug concentrations and interference with vitamin K-dependent clotting factor synthesis.^3,4^ Furthermore, there have been additional reports of coagulopathy with cefazolin and rifampin dual therapy when treating MSSA infections with prosthetic materials.^5^ However, reports of cefazolin monotherapy-induced coagulopathy remain limited.^6–9^ We describe a case of severe INR elevation following cefazolin initiation in a patient with MSSA bacteremia.

## Case presentation

**Figure 1.**
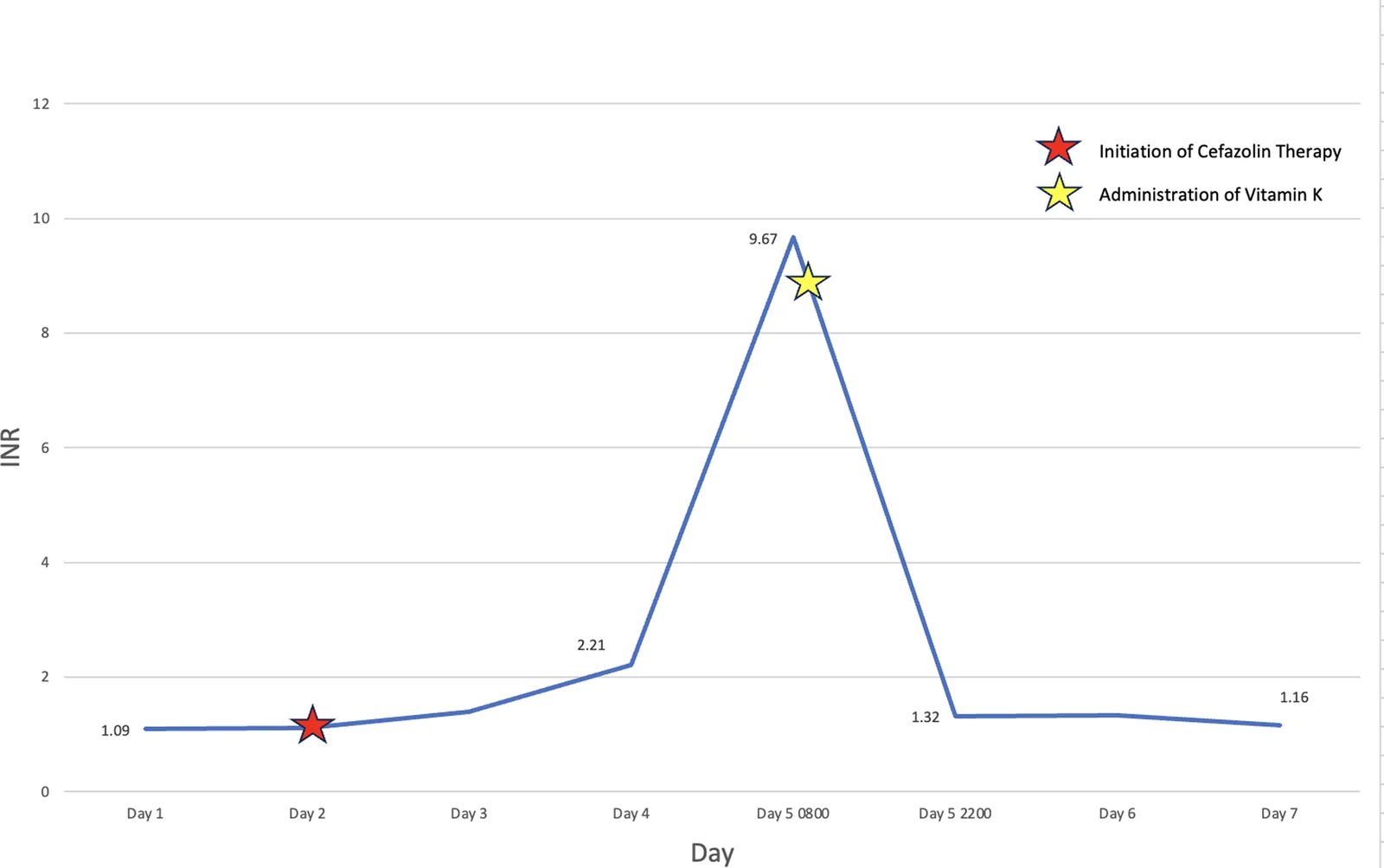
Figure 1 long description. INR trend over the hospital course relative to cefazolin initiation and vitamin K administration.

A 74-year-old male with a past medical history of hyperlipidemia, hypothyroidism, diabetes mellitus, prior gastrointestinal bleeding, NSTEMI, and chronic myeloid leukemia on dasatinib was admitted to the medical intensive care unit with septic shock secondary to a left arm skin and soft tissue infection. On admission, he was febrile (102.4°F) with leukocytosis (WBC 19.6 × 10^9^/L) and was started on vancomycin, piperacillin/tazobactam, and clindamycin. Blood cultures on hospital day 1 grew gram-positive cocci in clusters, with rapid diagnostics confirming MSSA. Of note, the patient had a severely elevated serum creatinine that reached levels almost four times his normal baseline requiring continuous renal replacement therapy and intermittent hemodialysis. Antibiotics were narrowed to cefazolin, and clindamycin was discontinued. At cefazolin initiation, his INR was 1.11. By day 3 of cefazolin therapy, the INR increased to 1.39 without an identifiable cause. During this time, he was also started on subcutaneous heparin for DVT prophylaxis and then transitioned to a heparin infusion for suspected acute coronary syndrome; however, this would not be expected to elevate INR. The INR continued to rise, reaching 7.09 on day 5 and >9.67 on repeat measurement (Figure 1). Ten millligrams of intravenous phytonadione was administered daily for 3 days to treat suspected coagulopathy and risk of bleeding, and the heparin infusion was held. Further evaluation revealed a prolonged PTT of 67 s, consistent with heparin therapy. In a heparinized patient, the PTT and anti-Xa should reflect an anticoagulated state together. Here, the anti-Xa was undetectable, indicating the absence of a meaningful heparin effect. The prolonged PTT therefore could not be attributed to heparin and instead reflected an underlying coagulopathy. Thromboelastography (TEG) showed a prolonged R time and decreased alpha angle consistent with clotting factor deficiency. Factor assays confirmed reduced activity of vitamin K-dependent clotting factors II, VII, IX, and X, consistent with vitamin K deficiency or antagonism. Factor V activity was also decreased. This finding is more consistent with ischemic hepatitis and suggests that hepatic dysfunction may have contributed to the coagulopathy. Following administration of vitamin K, the INR rapidly improved to 2.52 and subsequently normalized over several days, with repeat TEG demonstrating improved coagulation parameters. Cefazolin was discontinued, and therapy was completed with oxacillin, after which the INR remained stable. During this period, the patient also developed acute transaminase elevations, with AST peaking at 2,004 U/L and rapid subsequent improvement, findings consistent with ischemic hepatitis.

## Discussion

This case illustrates a rare but clinically significant instance of cefazolin-associated coagulopathy. The rapid rise in INR following cefazolin initiation, combined with decreased activity of vitamin K-dependent clotting factors and prompt correction with vitamin K, strongly supports a drug-induced mechanism. Cefazolin’s MTD side chain is hypothesized to inhibit vitamin K epoxide reductase, producing a warfarin-like effect that reduces synthesis of clotting factors II, VII, IX, and X.^1,2^ Although this phenomenon is well described with NMTT-containing cephalosporins, reports involving cefazolin remain sparse.^1,2,6,7^ In 2019, Strazzulla et al.^8^ conducted a retrospective chart review of all patients treated with cefazolin with monitored coagulation markers. They found that there was a slight increase in PTT in patients that received cefazolin, but no change in their INR. Additionally, bleeding only occurred in 5% of the total population. Ngiam et al.^9^ reported a cefazolin-associated INR elevation in a patient with MSSA endocarditis with end-stage renal disease (ESRD) that was also responsive to vitamin K therapy. Wang et al.^5^ published a case report detailing a patient with MSSA endocarditis that received cefazolin and rifampin dual therapy for treatment. This patient had a recent large weight loss and developed renal failure with a need for renal replacement therapy. It was thought that these events were provoking factors of cefazolin toxicity which may worsen coagulopathy. A 2026 case report by Beckstrom et al.^6^ described a similar occurrence to the one in this case report. The patient developed a seemingly unprecipitated INR increase that was rapidly corrected with 5 mg of oral vitamin K therapy. Finally, Smith et al.^7^ described another case of cefazolin-associated INR elevation in a patient with a MSSA infection. However, unlike other case reports, this patient only had a mild elevation in their serum creatinine compared to renal failure requiring renal replacement therapy. Application of the Naranjo Adverse Drug Reaction Probability Scale yielded a score of 4, consistent with a possible adverse drug reaction.^11^ Points were assigned for prior published reports, the temporal relationship to cefazolin initiation, improvement following discontinuation and phytonadione administration, and objective confirmation by factor assays and TEG, with a point deducted for the presence of concurrent ischemic hepatitis as an alternative cause. Several items could not be scored in this setting, including rechallenge, dose-response, and measurement of cefazolin concentrations, which are not routinely available. The presence of concurrent hepatic injury complicates interpretation. The patient’s marked transaminase elevation and rapid normalization are consistent with ischemic hepatitis. Importantly, hepatic synthetic dysfunction typically does not respond to vitamin K administration, as the liver is unable to produce new clotting factors.^10^ In contrast, this patient demonstrated a rapid INR correction following phytonadione, supporting a functional vitamin K deficiency rather than isolated hepatic failure. The reduction in factor V activity suggests that hepatic injury likely contributed to the overall coagulopathy. Therefore, this case is best explained by a multifactorial process, with cefazolin-induced inhibition of vitamin K-dependent clotting factor synthesis as the primary driver, compounded by transient hepatic dysfunction.

## Conclusion

Cefazolin-associated coagulopathy, though rare, should be considered in patients with unexplained INR elevation during therapy. Early recognition is critical, as discontinuation of the offending agent and administration of vitamin K can rapidly reverse coagulopathy. Clinicians should maintain heightened awareness in critically ill patients, particularly when additional risk factors for coagulopathy are present.

## Research transparency and reproducibility

Nothing to report.

## Figure 1. Long description

Line graph titled ‘Figure 1: Change in INR over the first 7 days of patient admission.’ The x-axis shows time points (Day 1, Day 2, Day 3, Day 4, Day 5 0800, Day 5 2200, Day 6, Day 7) and the y-axis shows INR from 0 to 12. INR begins at 1.09 on Day 1 and remains stable through Day 2 (1.09), when cefazolin therapy is initiated (marked with a red star). It rises gradually to 1.4 on Day 3 and 2.21 on Day 4, then sharply peaks at 9.67 on Day 5 0800, at which point vitamin K is administered (marked with a yellow star). INR then falls steeply to 1.32 by Day 5 2200 and stabilizes near baseline through Day 6 and Day 7 (1.16).

Navigate back to Figure 1..
